# Micro/Nanomechanical Characterization of ScAlMgO_4_ Single Crystal by Instrumented Indentation and Scratch Methods

**DOI:** 10.3390/ma17153811

**Published:** 2024-08-02

**Authors:** Zifeng Ni, Jie Yu, Guomei Chen, Mingjie Ji, Shanhua Qian, Da Bian, Ming Liu

**Affiliations:** 1 School of Mechanical Engineering, Jiangnan University, Wuxi 214122, China; 2 Jiangsu Province Engineering Research Center of Micro-Nano Additive and Subtractive Manufacturing, Wuxi 214122, China; 3 School of Electromechanical, Wuxi Vocational Institute of Commerce, Wuxi 214153, China; 4 Fujian Provincial Key Laboratory of Terahertz Functional Devices and Intelligent Sensing, School of Mechanical Engineering and Automation, Fuzhou University, Fuzhou 350108, China

**Keywords:** ScAlMgO_4_ single crystal, micromechanical properties, nanoindentation, microscratch, fracture toughness

## Abstract

ScAlMgO_4_ (SCAM), which can be used as an epitaxial substrate material of GaN in power devices, faces the challenge of achieving a high-quality surface by ultra-precision polishing due to its brittle and easily cleaved characteristics, which are closely associated with its mechanical properties. The micromechanical properties of SCAM single crystals were evaluated by nanoindentation and microscratch tests using different indenters. The elastic modulus *E*_IT_ and the indentation hardness *H*_IT_ of SCAM obtained by nanoindentation were 226 GPa and 12.1 GPa, respectively. Leaf-shaped chips and the associated step-like planes of SCAM can be found in the severely damaged regime during scratching by Berkovich and Vickers indenters with sharp edges due to the intersection of intense radial and lateral cracks. The fracture toughness (*K*_c_ = 1.12 MPa·m^1/2^) of SCAM can be obtained by using a scratch-based methodology for a spherical indenter based on linear elastic fracture mechanics (LEFM) under an appropriate range of applied loads. An optimal expression for calculating the fracture toughness of easily cleaved materials, including SCAM, via the Vickers indenter-induced cracking method using a Berkovich indenter was recommended.

## 1. Introduction

The lattice match between a semiconductor material and the epitaxial substrate (e.g., sapphire [1] and SiC [2]) is key to achieving performance breakthroughs in power devices made of semiconductor materials (e.g., GaN single crystals of a wide forbidden bandwidth and a high insulating breakdown field strength) [3]. ScAlMgO_4_ (SCAM) is the most promising epitaxial substrate of semiconductor GaN due to the small lattice mismatch (i.e., 1.8%) between SCAM and GaN, which has close coefficients in thermal expansion along the *a*-axis (6.2 × 10^−6^/K for GaN and 5.6 × 10^−6^/K for SCAM) [4,5]. The substrate material should be prepared by ultra-precision polishing, during which the micro-scale removal of material is achieved, and thus the microscopic removal mechanism and micromechanical properties of the material have crucial impacts on ultra-precision polishing [6], which can be simulated by instrumented scratch with the indenter serving as a single abrasive particle against the crystal surface [7]. Scratch tests can also be used to characterize the tribological behavior [8], material properties [9], and surface damage [10] of different materials (e.g., ceramics [11], glasses [12], and metals [13]). Instrumented indentation has been widely used to characterize the mechanical properties, including the elastic modulus [14], indentation hardness [15], and fracture toughness [16], of different materials.

Ge et al. [17] performed scratch tests on the (001) plane of silicon single crystal using a Berkovich indenter and found that edge-forward and face-forward scratching resulted in different residual depths and material removal rates. Hou et al. [18] performed indentation tests on the (0001) plane of 4H-SiC single crystal wafer and established an analytical expression of the total energy dissipated during a single indentation cycle. Feng et al. [19] carried out scratch tests on the (0001) plane (i.e., C-plane) of sapphire and reported that scratch speed had significant effects on surface damage, chip formation, and penetration depth. Using Vickers, Knoop, and Berkovich indenters under various loads, Liu et al. [20] investigated the mechanical properties (e.g., elastic modulus, indentation hardness, and fracture toughness) of ZrO_2_ and Si_3_N_4_ by indentation and scratch methods and found that mechanical properties of material, indenter geometry, and loading conditions all greatly affected the obtained values of fracture toughness depending on scratch methodology.

The high-quality surface of ScAlMgO_4_ prepared by ultra-precision polishing is challenging due to its brittle and easily cleaved characteristics, which are closely associated with its mechanical properties (mainly hardness and fracture toughness [21,22]) and which are lacking in study. To provide some guidance for ultra-precision polishing, in this study, the mechanical properties of the machining surface of SCAM were examined by microscratch and nanoindentation tests with different indenters. Indentation hardness and elastic modulus under different loads were compared and discussed via the nanoindentation technique through the OP method with a Berkovich indenter. In addition, we focus on the characterization of fracture toughness, which plays a critical role in the brittle fracture of SCAM. The values of fracture toughness obtained by different methods, including microscratch approaches with a spherical indenter and the Vickers indenter-induced cracking method with a Berkovich indenter, were compared and discussed.

## 2. Materials and Experimental Methods

### 2.1. SCAM Wafer

**Figure 1:**
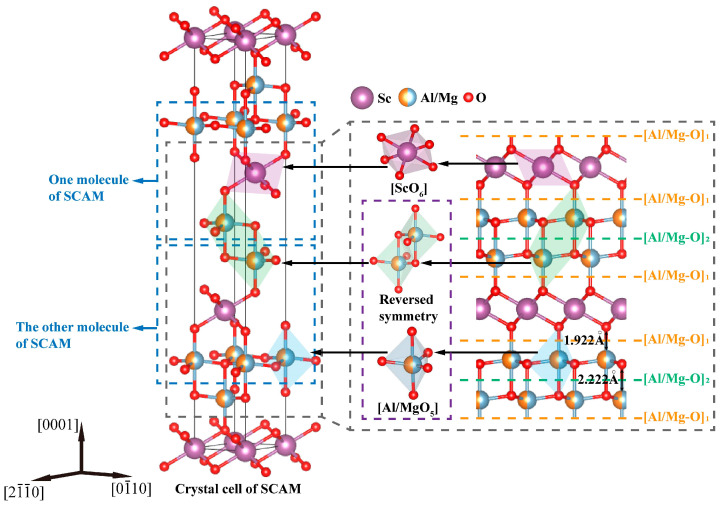
Crystal cell (lattice parameters: a = b = 0.324 nm, c = 2.515 nm) of a SCAM single crystal with three SCAM molecules. Two types of cleavage surfaces are represented by [Al/Mg-O]_1_ and [Al/Mg-O]_2_.

SCAM has a crystal structure of YbFe_2_O_4_ and consists of a hexagonal crystal system with the $R\bar{3}m$ spatial group, with each crystal cell containing three SCAM molecules, as shown in Figure 1. Sc^3+^ occupies the position of Yb in the YbFe_2_O_4_ crystal structure, forming an octahedral structure with six O^2−^ ions, and Al^3+^/Mg^2+^ randomly occupies the position of Fe in the YbFe_2_O_4_ crystal structure, forming an [Al/MgO_5_] triangular bipyramid structure of five O^2−^ ions [23]. SCAM is considered a type of (RAO_3_)_n_(MO)_m_ group oxide (n = m = 1 for SCAM; R^3+^ indicates rare-earth metallic elements such as In/Lu/Y/SC; A^3+^ can consist of metallic elements such as Fe/Ga/Al; and M^2+^ can consist of metallic elements such as Mg/Co/Cu/Zn) [24]. SCAM tends to cleave along the (0001) plane, making it challenging to obtain a high-quality wafer surface. Two types of Al/Mg-O bonds will result in two different (0001) planes, as represented by [Al/Mg-O]_1_ and [Al/Mg-O]_2_, respectively. SCAM can cleave at [Al/Mg-O]_1_, mainly due to weak interconnections between the layer of [ScO_6_] and [Al/MgO_5_] by O^2-^ irons [25]. SCAM can also cleave at [Al/Mg-O]_2_, mainly due to the reverse symmetry that can cancel the electric dipole moment and surface charges [26].

**Figure 2:**
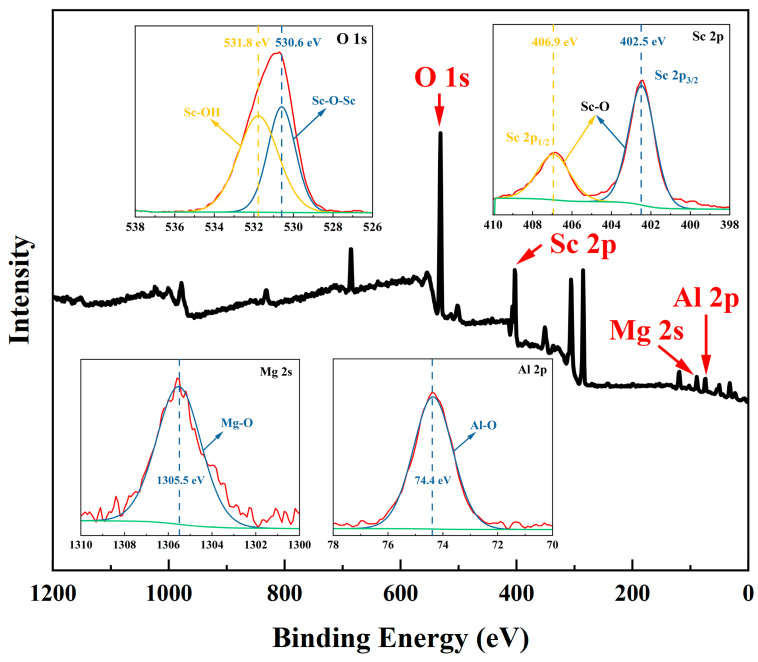
X-ray photoelectron spectroscopy spectra of the SCAM wafer before polishing. The peaks of Sc 2p, Mg 2s, Al 2p, and O 1s were calibrated with C 1s (284.4 eV) as the standard. The insets show the high-resolution spectrogram of the elements Sc 2p, Al 2p, Mg 2s, and O 1s, with peak fitting by Avantage software (Ver. 6.6.0).

Figure 2 shows the X-ray photoelectron spectroscopy (XPS, EscaLab 250Xi, Thermo Fisher, Waltham, MA, USA) of the SCAM wafer with the following dimensions: 10 × 10 × 0.5 mm^3^ (Goetsu Semiconductor WuXi Co., Ltd., Wuxi, China) before polishing. The fine spectra of Sc 2p, Mg 2s, Al 2p, and O 1s of SCAM are also shown as insets. The Sc 2p of SCAM corresponds to the Sc-O bond with two different binding energies of 402.5 eV and 406.9 eV, respectively [27]. Mg 2s and Al 2p correspond to Mg-O (binding energy of 1305.5 eV) and Al-O (binding energy of 74.4 eV) bonds, respectively [28,29]. The peaks of O 1s at 530.6 eV and 531.8 eV correspond to the Sc-O-Sc and Sc-OH bonds (due to water in air), respectively [30].

Colloidal SiO_2_ particles about 100 nm in size (Wujiang Chuangyuan New Material Technology Co., Ltd., Suzhou, China) were used as the polishing slurry. Potassium hydroxide KOH (Sinopharm Chemical Reagent Co., Ltd., Beijing, China) was selected as a pH regulator. SCAM wafers were polished under a pH of 8, an abrasive particle concentration of 20 wt.%, and a slurry flow rate of 200 mL/min on JY-M15P (Jeng Yueh Enterprise Co., Ltd., New Taipei City, Taiwan) to ensure the surface quality for subsequent nanoindentation and microscratch tests. The polished wafers were ultrasonically cleaned with anhydrous ethanol and deionized water, and the surfaces were blow-dried using an air gun.

**Figure 3:**
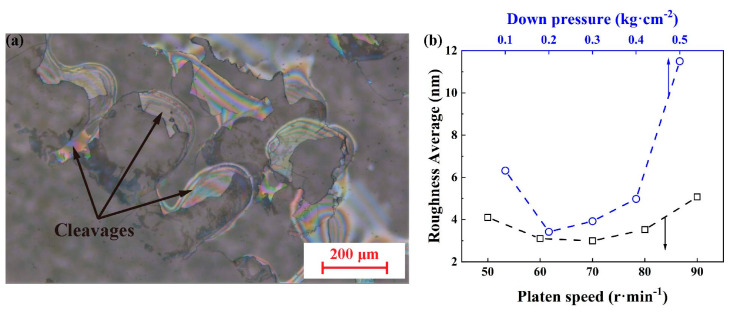
(**a**) Metallographic microscope image (BH200M, Sunny Optical Technology Group Co., Ltd., Yuyao, China) of SCAM surface after polishing under platen speed of 80 r/min and down pressure of 0.5 kg/cm^2^, (**b**) effects of down pressure (under a fixed platen speed of 80 r/min) and platen speed (under a fixed down pressure of 0.2 kg/cm^2^) on roughness average *R*_a_.

Figure 3a shows the metallographic microscope image of the SCAM surface after polishing with a down pressure of 0.5 kg/cm^2^ and a platen speed of 80 r/min. Cleavages appear as colored waves due to the large down pressure of 0.5 kg/cm^2^ applied for polishing. Figure 3b shows the effects of the polishing down pressure (under a fixed platen speed of 80 r/min) and a platen speed (under a fixed polishing down pressure of 0.2 kg/cm^2^) on the roughness average *R*_a_ measured by atomic force microscopy (scan area of 10 × 10 μm^2^). The down pressure and platen speed should be optimized to obtain the lowest *R*_a_. A sharp increase in *R*_a_ occurs as the down pressure increases to 0.5 kg/cm^2^, which is the reason for the formation of cleavages shown in Figure 3a. The down pressure has a greater influence on *R*_a_ than platen speed. The optimal polishing conditions for the lowest *R*_a_ can be determined to be a down pressure of 0.2~0.3 kg/cm^2^ and a platen speed of 60~80 r/min.

**Figure 4:**
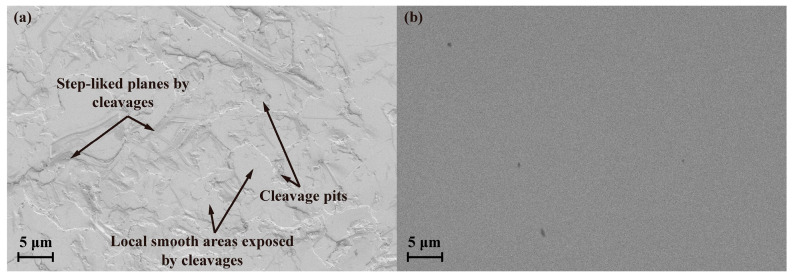
Scanning electron microscopy (SIGMA-500, ZEISS, Oberkochen, German) images of SCAM surfaces: (**a**) before polishing; and (**b**) after polishing with platen speed of 80 r/min and down pressure of 0.2 kg/cm^2^.

Figure 4a,b compare the scanning electron microscopy (SEM) images of the SCAM surfaces before and after polishing under a platen speed of 80 r/min and a down pressure of 0.2 kg/cm^2^. Step-like cleavage planes, cleavage pits, and local smooth areas exposed by cleavages can be observed on the initial surface of the easily cleaved SCAM, as shown in Figure 4a. The rough surface of the SCAM wafer becomes smooth and without cleavages after polishing under optimized conditions, and the polished surfaces are suitable for subsequent nanoindentation and microscratch tests.

### 2.2. Microscratch of SCAM

**Figure 5:**
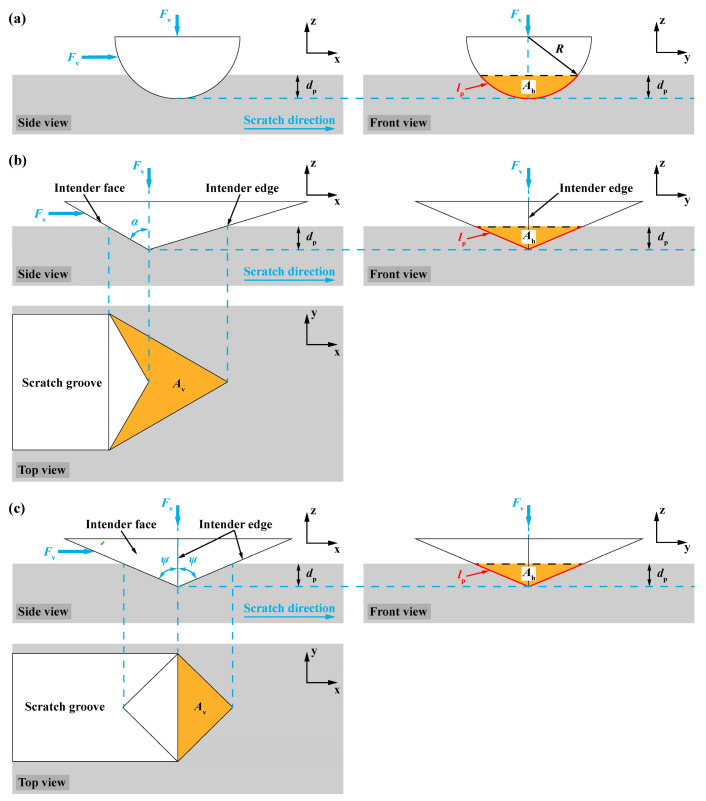
Schematic illustration of scratching by (**a**) spherical, (**b**) Berkovich and (**c**) Vickers indenters.

Instrument scratch tests were performed on an MST^2^ (Anton Paar, Graz, Austria) microscratch tester equipped with acoustic emission (AE) [31], and three different types of indenters were used, namely a diamond Berkovich indenter with a face angle of 65.27° (resulting in the same ratio of the projected contact area over the contact depth as the Vickers indenter) [32], a diamond Vickers indenter, and a spherical indenter made of steel (i.e., 100Cr6) with a radius of 500 μm. Tests were conducted on the plane that had a deviation of 16°12′ degrees from the (0001) plane (i.e., the 10 × 10 mm^2^ surface of the wafer) of SCAM with a scratch direction parallel to one side of the 10 × 10 mm^2^ surface. The scratch distance was 3 mm, and the thrust was in the edge-forward direction for the sharp indenters. The normal load *F*_v_ was progressively increased from the initial 5 mN to three different maximum loads (i.e., 0.5 N, 5 N, and 25 N) within 30 s in the scratch tests with a spherical indenter at a scratch speed of 6 mm/min. Four different scratch speeds (i.e., 3, 6, 12, and 18 mm/min) were applied in the microscratch tests using Berkovich and Vickers indenters, and the normal load *F*_v_ was progressively increased from the initial 5 mN to a maximum of 500 mN. Since the scratch speed was much lower than the processing speeds during grinding and polishing, the thermal effect was negligible. The scratch test consisted of four steps [33]: (1) the initial surface profile or sample tilt of the SCAM wafer was measured during the pre-scan phase under a small vertical load of 5 mN, whose effect on plastic deformation was negligible [34]; (2) scratching under the prescribed conditions; (3) post-scan under a small vertical load of 5 mN, and the residual depth could be obtained; and (4) capture of scratch morphology by optical microscopy [35]. The residual scratch morphologies obtained by optical microscopy were synchronized with the variations of scratch variables, such as penetration depth *d*_p_ representing total deformation, residual depth *d*_r_ representing plastic deformation, friction coefficient *μ* (i.e., the ratio of lateral force over normal load), and acoustic emission (AE) during scratching as *F*_v_ increased [36]. The horizontally projected contact area *A*_h_ and the vertically projected contact area *A*_v_ for different indenters are illustrated in Figure 5 and [37]:

(1) $$A_{h}=\left\{ \begin{array}{l} \sqrt{3}d_{p}^{2}\tan\alpha \;\mathrm{for}\;\mathrm{Berkovich}\;\mathrm{indenter},\;\mathrm{and}\;\alpha =65.27{}^{\circ}\\ \sqrt{2}d_{p}^{2}\tan\psi \;\mathrm{for}\;\mathrm{Vickers}\;\mathrm{indenter},\;\mathrm{and}\;\psi =68{}^{\circ}\\ R^{2}\arccos\frac{R-d_{p}}{R}-(R-d_{p})\sqrt{R^{2}-{\left(R-d_{p}\right)}^{2}}\;\mathrm{for}\;\mathrm{spherical}\;\mathrm{indenter} \end{array}\right.$$

(2) $$A_{v}=\left\{ \begin{array}{l} 2\sqrt{3}d_{p}^{2}\tan\alpha \;fοr\;\mathrm{Berkovich}\;\mathrm{indenter},\;\mathrm{and}\;\alpha =65.27{}^{\circ}\\ 2d_{p}^{2}\tan\psi \;\mathrm{for}\;\mathrm{Vickers}\;\mathrm{indenter},\;\mathrm{and}\;\psi =68{}^{\circ} \end{array}\right.$$

### 2.3. Nanoindentation of SCAM

Instrumented indentation tests of SCAM were performed on a NHT^2^ (Anton Parr) nanoindentation tester by a Berkovich indenter with a face angle of 65.27° under both the loading and unloading times of 30 s, a holding time of 10 s, and a sampling frequency of 10 Hz. Indentation tests were conducted on the same surface and in the same direction as the scratch tests. Both loading and unloading segments of the indentation load-displacement curve can be fitted by simple power law functions as follows [38]:

(3) $$F=\left\{ \begin{array}{l} k_{1}h^{n_{1}}\;\mathrm{for}\;\mathrm{loading}\\ F_{\max}(\frac{h-h_{p}}{h_{\max}-h_{p}}{)}^{m}\;\mathrm{for}\;\mathrm{unloading} \end{array}\right.$$

where *k*_1_ and n_1_ are fitting parameters; *F* and *h* are indentation load and displacement, respectively, with the subscript “max” indicating the maximum value; *m* is an exponent ranging between 1.2 and 1.7 for most materials [39,40]; and *h*_p_ is the permanent indentation displacement (i.e., the residual indentation displacement after unloading).

The reduced plane strain modulus *E*_r_ of the contact area is a combination of the plane strain modulus of the sample and that of the indenter and can be obtained from the contact stiffness *S* [41] (i.e., the initial slope of the unloading curve at *h*_max_):

(4) $$E_{r}=\frac{1}{\left(\frac{1-\nu^{2}}{E_{\mathrm{IT}}}+\frac{1-\nu_{i}^{2}}{E_{i}}\right)}=\frac{\sqrt{\pi }S}{2\beta_{1}\sqrt{A_{p}(h_{c})}},\;S={\frac{dP}{dh}|}_{h_{\max}}$$

where *E*_IT_ and *ν* denote the elastic modulus and Poisson’s ratio (*ν* = 0.2 is used for SCAM) of the sample, respectively; and *E*_i_ = 1141 GPa and *ν*_i_ = 0.07 are the elastic modulus and Poisson’s ratio of the diamond indenter, respectively. *β*_1_ = 1.034 is the correction factor for a Berkovich indenter lacking axial symmetry [41].

Both indentation hardness *H*_IT_, which is the mean projected contact pressure, and contact depth *h*_c_ can be obtained at *h*_max_:

(5) $$H_{IT}=\frac{F_{\max}}{A_{p}(h_{c})},h_{c}=h_{\max}-\varepsilon \frac{F_{\max}}{S}$$

where *A*_p_ is the projected contact area at *h*_max_. Since the calibration of indenters is the basis for obtaining accurate mechanical properties of tested materials via the OP method [42], the *A*_p_(*h*_c_) of the Berkovich indenter was calibrated by performing nanoindentation tests on a standard fused silica with known elastic modulus of 73 GPa and a Poisson’s ratio of 0.17 under various loads (≤100 mN for fused silica in order to avoid cracking) with B-spline interpolation in ambient laboratory condition, and ε depends on the value of m [43].

## 3. Results and Discussion

### 3.1. Analysis of Nanoindentation of SCAM by a Berkovich Indenter

**Figure 6:**
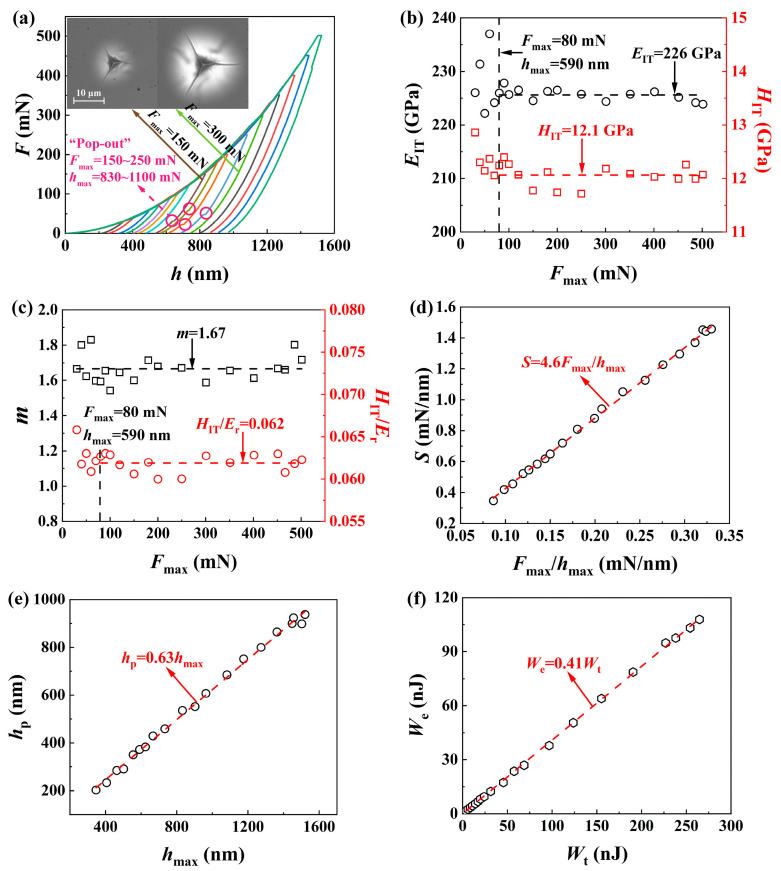
Results of nanoindentation of SCAM with a Berkovich indenter under different loads: (**a**) *F*-*h* curves (the insets show the residual imprints at *F*_max_ = 150 mN and 300 mN); variations of (**b**) *H*_IT_ and *E*_IT_, and (**c**) m and *H*_IT_/*E*_r_ with *F*_max_; linear relationships between (**d**) *S* and *F*_max_/*h*_max_, (**e**) *h*_p_ and *h*_max_, and (**f**) *W*_e_ and *W*_t_.

Figure 6a shows the load-displacement (*F*-*h*) curves of SCAM under different loads of *F*_max_ for SCAM, and the creep effect during the holding period is negligibly small for brittle solids. The elastic recovery during the unloading indicated that both elastic deformation and plastic deformation played significant roles during the indentation of hard and brittle SCAM at the nanoscale. When *F*_max_ = 150 mN, radial cracks and the “pop-out” phenomenon (i.e., a reverse small jump in the penetration depth during unloading) were observed, which could be attributed to the cleavage fracture of SCAM. The “pop-out” phenomenon was sensitive to the applied load and disappeared under large loads (i.e., *F*_max_ > 300 mN) when complex cracking systems were generated on the subsurface region, under which conditions cleavage fractures played a less significant role in indentation-induced damage.

Figure 6b shows variations of nanoindentation hardness *H*_IT_ and elastic modulus *E*_IT_ with *F*_max_. The decrease in *H*_IT_ and *E*_IT_ with increasing *F*_max_ under small loads (i.e., *F*_max_ < 80 mN) could be attributed to residual stresses after polishing. Both *H*_IT_ and *E*_IT_ reached constant levels (i.e., *H*_IT_ = 12.1 GPa, *E*_IT_ = 226 GPa) when *F*_max_ ≥ 80 mN, indicating that *H*_IT_ and *E*_IT_ of SCAM were insensitive to indentation-induced damage (i.e., cleavage fracture and radial cracks).

Figure 6c shows variations of *m* and *H*_IT_/*E*_r_ with *F*_max_. A constant *m* of 1.67 could be approximated for SCAM. *H*_IT_/*E*_r_ tended to decrease as the load increased under small loads (i.e., *F*_max_ < 80 mN), which could be attributed to elastic deformation and surface effect. A constant *H*_IT_/*E*_r_ of 0.062 was calculated for SCAM under large loads and was smaller than those of brittle glass (i.e., *H*_IT_/*E*_r_ = 0.10 for K9 glass [40] and *H*_IT_/*E*_r_ = 0.13 for fused silica [41]), demonstrating that SCAM is less brittle than glass.

Figure 6d shows the proportional relationship between the contact stiffness *S* and *F*_max_/*h*_max_, which could be used to continuously measure *S* with the load and displacement measured during the loading segment without the need of unloading, resulting in determining both *H*_IT_ and *E*_IT_ under different loads (or penetration depths) from only one loading test [44].

Figure 6e shows that the permanent depth *h*_p_ is also proportional to the maximum indentation displacement *h*_max_. *h*_p_/*h*_max_ = 0.63 for SCAM is smaller than 0.7, indicating the absence of pile-up [45], which was confirmed by the insets in Figure 6a. *h*_p_/*h*_max_ = 0.63 for SCAM is larger than those of fused silica (i.e., 0.48) [41] and K9 glass (i.e., 0.55) [40], which also indicates SCAM is less brittle than glass.

Figure 6f shows that elastic deformation work *W*_e_, which can be calculated by integrating the unloading load-displacement curve, is proportional to the total mechanical work *W*_t_ of the indentation, which can be calculated by integrating both the loading and holding segments of the load-displacement curve. *W*_e_/*W*_t_ was about 41% for SCAM due to the prominent roles of both elastic deformation and plastic deformation during nanoindentation of brittle material. *W*_e_/*W*_t_ of SCAM was smaller than those of fused silica (i.e., 0.65) [41] and K9 glass (i.e., 0.55) [40], which also indicates SCAM is less brittle than glass.

**Figure 7:**
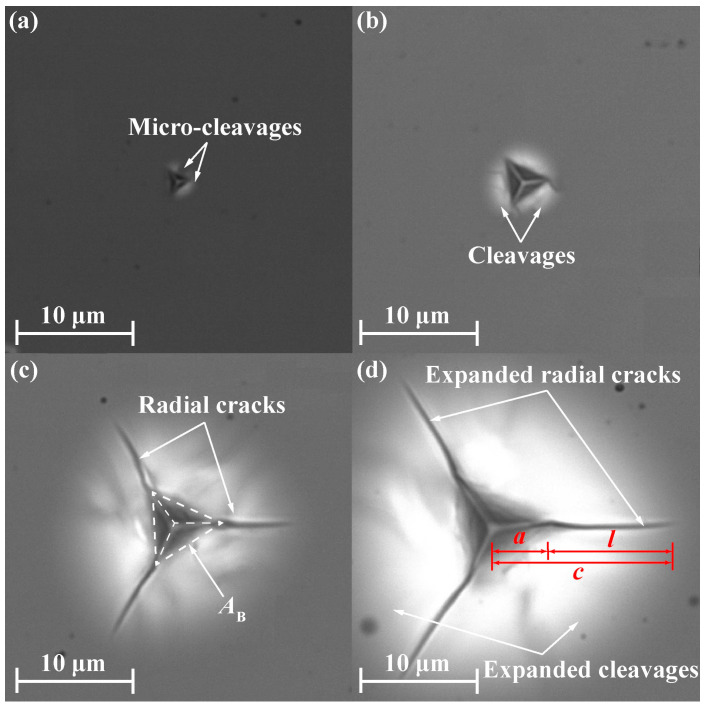
Residual imprints and different forms of damage after nanoindentation tests of SCAM by Berkovich indenter under different loads: (**a**) *F*_max_ = 30 mN, (**b**) *F*_max_ = 100 mN, (**c**) *F*_max_ = 250 mN (the project area of the imprint by Berkovich indenter *A*_B_ is highlighted by the dotted lines), and (**d**) *F*_max_ = 400 mN (*c* is the radius of radial crack measured from the imprint center, *l* is the surface crack length for radial cracking, and *a* = *c* − *l*).

The residual indentation morphologies of SCAM at four different loads are shown in Figure 7a–d. Only micro-cleavages were found on the wafer surface under small loads, see Figure 7a for *F*_max_ = 30 mN. Obvious cleavages were observed at the edges of the residual imprint under relatively large loads, see Figure 7b for *F*_max_ = 100 mN. Radial cracks initiated from the three vertices of residual imprints by the Berkovich indenter under sufficiently large loads, see Figure 7c for *F*_max_ = 250 mN. As *F*_max_ continued to increase, radial cracks and cleavages expanded, see Figure 7d for *F*_max_ = 400 mN.

### 3.2. Analysis of Microscratch of SCAM by Different Indenters

**Figure 8:**
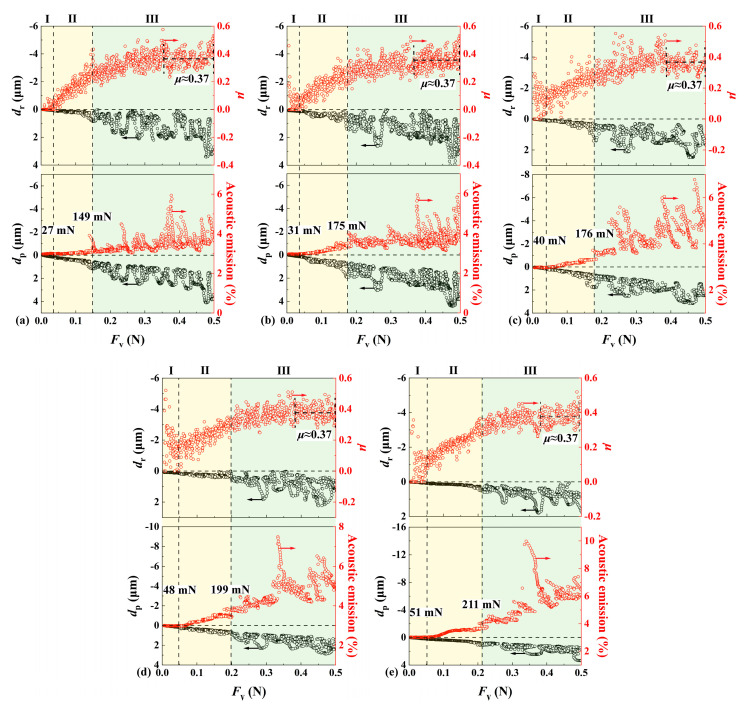
Variations of scratch variables with the applied normal load *F*_v_ during scratching by a Berkovich indenter under a progressive load linearly increasing from 5 mN to 500 mN and different scratch speeds: (**a**) 3 mm/min; (**b**) 6 mm/min; (**c**) 9 mm/min; (**d**) 12 mm/min; and (**e**) 18 mm/min. The critical loads indicating the two transitions from regime I to regime II, and from regime II to regime III, respectively, are denoted by vertical dotted lines.

**Figure 9:**
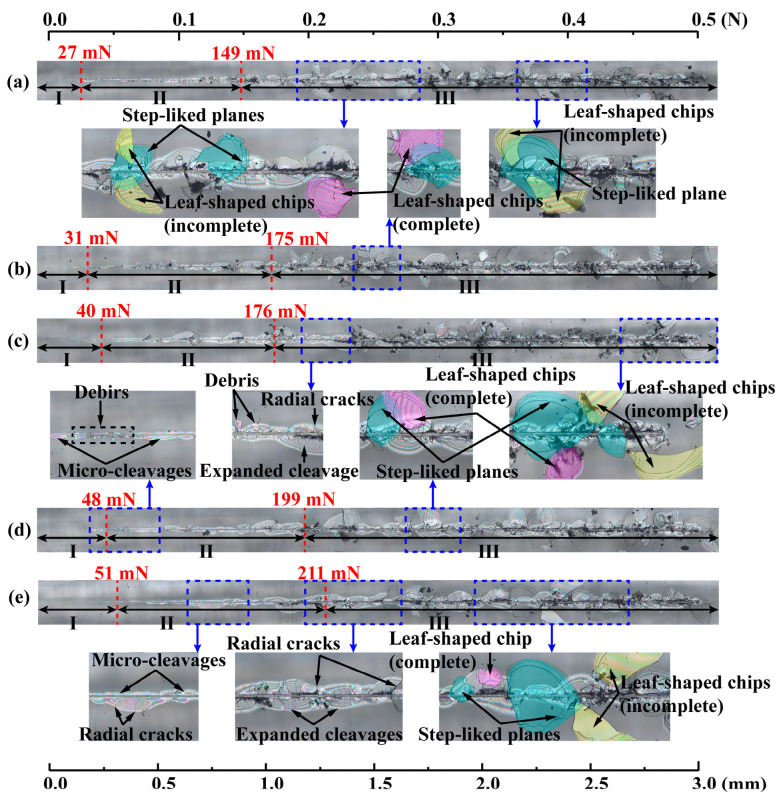
Optical images of residual scratch morphologies by a Berkovich indenter under progressively increasing load from 5 mN to 500 mN and different scratch speeds: (**a**) 3 mm/min; (**b**) 6 mm/min; (**c**) 9 mm/min; (**d**) 12 mm/min; (**e**) 18 mm/min. The step-like planes, marked by a blue color, are generated by the peeling off of complete (purple color) and incomplete (yellow color) leaf-shaped chips. The critical loads *F*_c1_ and *F*_c2_ determined by the variations of scratch variables in Figure 8 are displayed as red dotted lines.

Figure 8a–e show the variations of scratch variables including *d*_p_, residual depth *d*_r_, friction coefficient *μ*, and acoustic emission (AE) with the applied normal load *F*_v_ by a Berkovich indenter under progressive load that linearly increased from 5 mN to 500 mN and with different scratch speeds (i.e., 3, 6, 9, 12, and 18 mm/min). Figure 9a–e show the corresponding residual scratch morphologies that were synchronized with the scratch variables. Three different regimes were identified based on the variations of the scratch variables. In regime I under small loads, AE remained at low levels; friction coefficient *μ* vibrated around a low level; *d*_p_ increased in an approximately linear and smooth way with increasing *F*_v_; *d*_r_ was negligible; and micro-cleavages were seen in the absence of cracking. The transition from regime I to regime II was denoted by the clearly increasing trends of *μ* and AE. In regime II under intermediate loads, both AE and *μ* increased with the increase in *F*_v_; vibrations in *d*_r_ and *d*_p_ became prominent; the continuous appearance of micro-cleavages with their expansion was seen; debris, as well as lateral and radial cracks, appeared in the absence of leaf-shaped chips. The transition from regime II to regime III was denoted by the abrupt change in *d*_r_ and *d*_p_, together with the sharp increase in AE. In regime III under large loads, the increasing trend of *μ* gradually slowed down and vibrated around 0.37; significant fluctuations in *d*_p_, *d*_r_, and AE occurred; lateral and radial cracks intensively and continuously appeared; the step-like planes marked by blue color were generated by the peeling off of leaf-shaped chips marked in purple in Figure 9; and the expanded cleavages of a ripple shape similar to the cleavages by polishing as shown in Figure 3a were clearly seen. Both complete and incomplete leaf-shaped chips were seen and marked by purple and yellow colors, respectively, in Figure 9: one step-like plane was associated with two incomplete leaf-shaped chips or one complete leaf-shaped chip. The range in regime I was very small compared with those of regimes II and III, indicating the brittle characteristics of SCAM.

**Figure 10:**
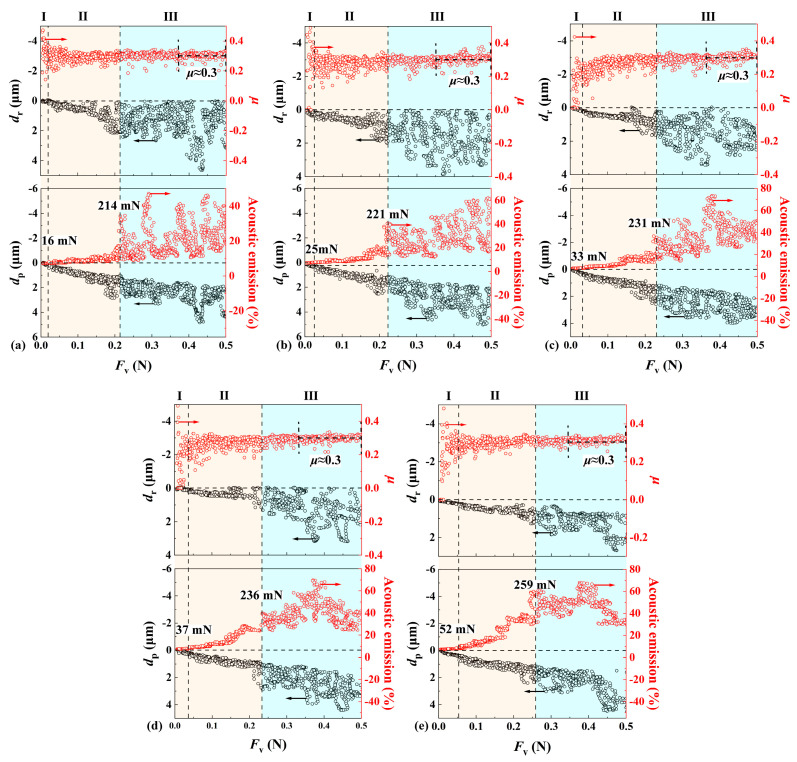
Variations of scratch variables with an applied normal load *F*_v_ during scratching by Vickers indenter under progressive, linearly increasing load from 5 mN to 500 mN and different scratch speeds: (**a**) 3 mm/min; (**b**) 6 mm/min; (**c**) 9 mm/min; (**d**) 12 mm/min; and (**e**) 18 mm/min. The critical loads indicating the two transitions from regime I to regime II, and from regime II to regime III, respectively, are denoted by vertical dotted lines.

**Figure 11:**
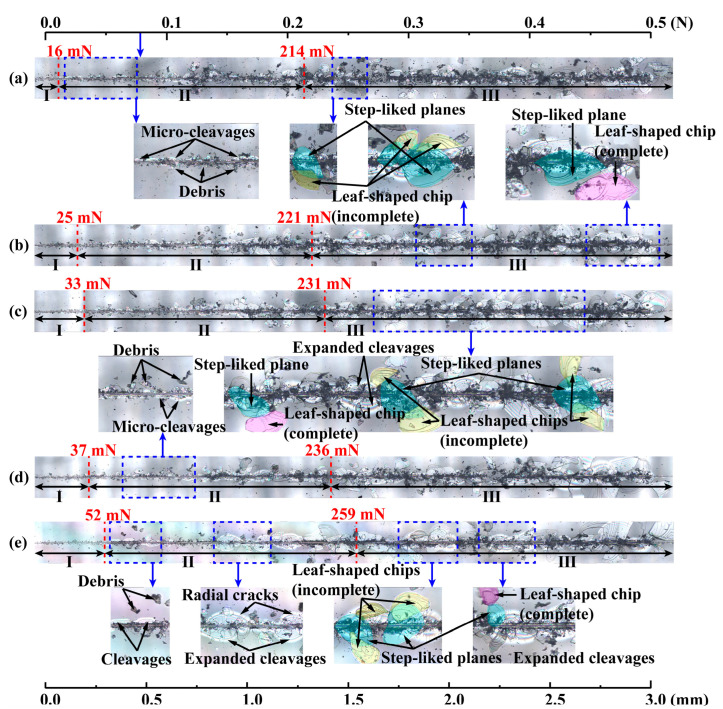
Optical images of residual scratch morphologies by a Vickers indenter under a linearly increasing load from 5 mN to 500 mN and different scratch speeds: (**a**) 3 mm/min; (**b**) 6 mm/min; (**c**) 9 mm/min; (**d**) 12 mm/min; (**e**) 18 mm/min. The step-like planes, marked by a blue color, are generated by peeling off of complete (purple color) and incomplete (yellow color) leaf-shaped chips. The critical loads *F*_c1_ and *F*_c2_ determined by the variations of scratch variables in Figure 10 are displayed as red dotted lines.

Figure 10a–e show the variations of scratch variables with the applied load *F*_v_ by a Vickers indenter under a linearly increasing load from 5 mN to 500 mN and different scratch speeds (i.e., 3, 6, 9, 12, and 18 mm/min). Three different regimes were identified based on the variations of scratch variables, which were synchronized with the residual groove morphologies as shown in Figure 11a–e. In regime I under small loads, *μ* vibrated significantly; AE intensity remained at a low level; *d*_r_ was almost zero; and *d*_p_ increased with increasing *F*_v_ in an approximately linear way; debris was seen, while micro-cleavages or radial cracks were hardly observed. The transition from regime I to regime II was denoted by the stabilization of *μ* and a clearly increasing trend of both AE and *d*_r_. In regime II under intermediate loads, *μ* stabilized at around 0.3; AE kept increasing with no abrupt change; both *d*_r_ and *d*_p_ increased with *F*_v_ in an almost linear way; and micro-cleavages, radial and lateral cracks, leaf-shaped chips, and step-like planes were observed. The transition from regime II to regime III was denoted by the abrupt increase in AE, which was associated with the large fluctuation of *d*_r_ spanning from zero to the value that deviated far from the linear trend. The debris accumulated in the scratch groove had a significant effect on *d*_r_, resulting its fluctuation and value of almost zero [40]. In regime III under large loads, *μ* vibrated within a smaller range of around 0.3; significant fluctuations in *d*_r_, *d*_p_ and AE occurred due to severe damage; continuously and intensively expanded cleavages, large radial and lateral cracks, leaf-shaped chips, step-like planes, and a large amount of debris was seen. With a Vickers indenter, *μ* vibrated significantly under small loads, and stabilized at about 0.3 under intermediate loads, while *μ* with a Berkovich indenter vibrated, kept increasing under intermediate loads, and reached a constant level of about 0.37 under large loads. The increase in *μ* was partly attributed to the interaction between leaf-shaped chips and indenter, and a larger *μ* was associated with more leaf-shaped chips [46]. Under large loads, leaf-shaped chips were destroyed and turned into debris [47], resulting in a constant level of *μ*. Although Berkovich (face angle of 65.27°) and Vickers (face angle of 68°) indenters can be regarded to be equivalent for indentation, a Vickers indenter can cause more serious damage compared to a Berkovich indenter, since the two sharp edges of a Vickers indenter that are perpendicular to the scratch direction can exert more squeeze on the sample. Three of the four sharp edges of a Vickers indenter play significant roles in scratching, while two of the three sharp edges of a Berkovich indenter cannot play an effective cutting role as one thrusts the material in front of the indenter. Therefore, a considerable amount of leaf-shaped chips can be turned into debris under smaller loads by a Vickers indenter, resulting in an earlier entry of *μ* into a stable level compared with a Berkovich indenter.

**Figure 12:**
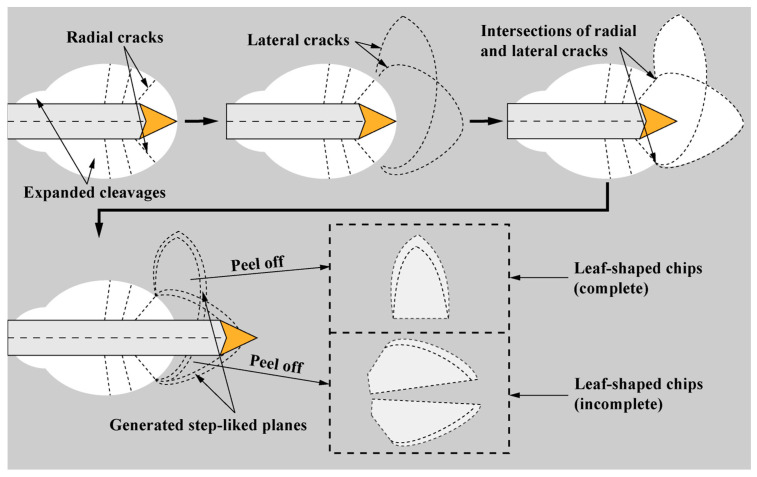
Schematic illustration (top view) of the mechanism of generation of leaf-shaped chips and corresponding step-like planes during scratching by the sharp indenter (Berkovich indenter is shown).

The generation mechanism of leaf-shaped chips and the corresponding step-like planes by a sharp indenter (e.g., Berkovich and Vickers indenters) is shown in Figure 12. Radial cracks, which occur together with the expanded cleavages, can intersect with lateral cracks that extend upwards to the surface of the sample under large loads [7], forming leaf-shaped areas to be delaminated (i.e., generation of leaf-shaped chips) due to the easily cleaved characteristics of SCAM. When the sharp indenter scratched through the leaf-shaped area in front of the indenter, the leaf-shaped chip was divided into two halves. When the leaf-shaped area was generated on the side of the scratch, and was peeled off from the sample, a complete leaf-shaped chip was generated. The step-like planes were exposed fresh surfaces after peeling off of the leaf-shaped chips.

In addition, according to the adhesion-ploughing friction theory, the friction force between two contact surfaces can be split into two parts: adhesion friction and ploughing friction [48]. Accordingly, the experimentally measured scratch friction coefficient *μ* can be divided into the adhesion friction coefficient *μ*_a_ and the ploughing friction coefficient *μ*_p_:

(6) $$\mu =\mu_{a}+\mu_{p},\mu_{\mathrm{pB}}=\frac{A_{hB}}{A_{vB}},\mu_{\mathrm{pV}}=\frac{A_{hV}}{A_{vV}},\mu_{a}=\mu -\mu_{p}$$

where the subscripts “B” and “V” indicate Berkovich and Vickers indenters, respectively; *μ*_pB_ and *μ*_pV_ are ploughing friction coefficients for Berkovich and Vickers indenters, respectively, and can be computed with Equations (1) and (2). A value of *μ*_pV_ = 0.29 is almost the same as a value of *μ* = 0.3 for a Vickers indenter under large loads, indicating the predominant role of brittle fractures with severe damage and negligible plasticity of the material. A value of *μ*_pB_ = 0.23 is much smaller than a value of *μ* = 0.37 for a Berkovich indenter under large loads, which can be explained by noting that two of the three sharp edges of the Berkovich indenter are more capable of generating pile-up than causing damage.

**Figure 13:**
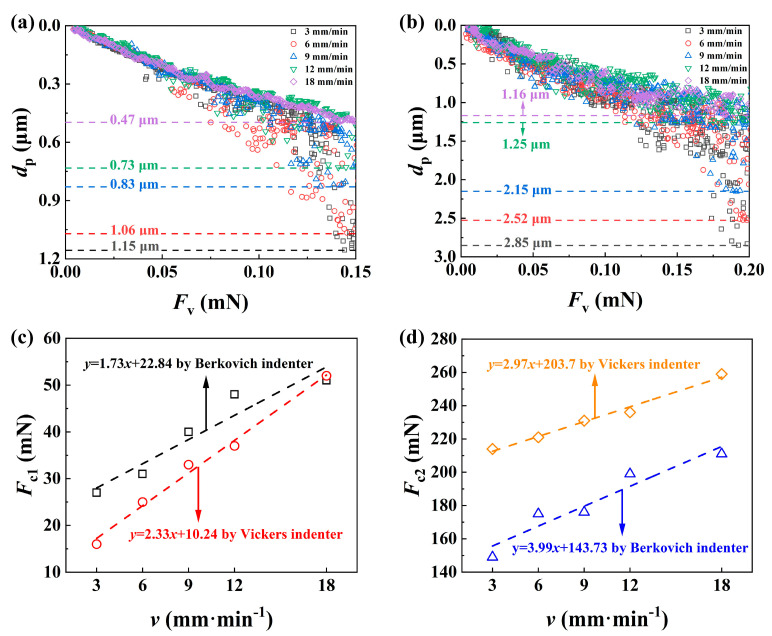
Effects of scratch speed on penetration depth *d*_p_ for Berkovich (**a**) and Vickers (**b**) indenters, and two critical loads *F*_c1_ (**c**) and *F*_c2_ (**d**).

Figure 13a,b compare the penetration depth *d*_p_ of SCAM under different scratch speeds using the Berkovich indenter under the maximum applied *F*_v_ = 150 mN and using the Vickers indenter under the maximum applied *F*_v_ = 200 mN, respectively, since the data of *d*_p_ become very scattered under large loads due to severe damage. A higher *v* tends to produce a smaller *d*_p_, which has been widely reported in the literature, and increasing the scratch speed can reduce the penetration ability of the indenter [49,50], resulting in a greater applied load for the same penetration effect under a lower scratch speed. Figure 13c,d show the effects of scratch speed on the two critical loads *F*_c1_ and *F*_c2_, respectively, and both *F*_c1_ and *F*_c2_ increased in an approximately linear way as the scratch speed increased for both indenters, since a larger *v* resulted in a smaller *d*_p_ and thus less damage, which corroborates the fact that increasing the platen speed within a certain range during polishing can reduce *R*_a_ of the material, see Figure 3b.

**Figure 14:**
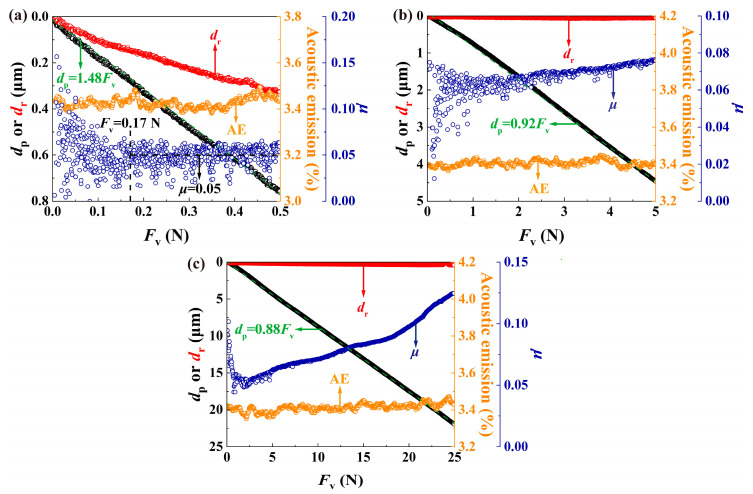
Variations of scratch variables during the scratch test by a spherical indenter under three progressively increasing normal loads: (**a**) 5~500 mN; (**b**) 5 mN~5 N; and (**c**) 5 mN~25 mN.

Figure 14a–c show the variations of scratch variables with the applied vertical load *F*_v_ during scratching by a spherical intender under three progressive loads (i.e., 5~500 mN, 5 mN~5 N, and 5 mN~25 N). *d*_p_ increased with increasing *F*_v_ in an approximately linear way, which has been widely reported in the literature for different materials (i.e., ceramic, copper, and glasses) [47,51,52], and the proportional coefficient of *d*_p_ against *F*_v_ decreased as the range of *F*_v_ increased due to the more prominent increase in contact area. AE intensity stabilized at about 3.4%, and values of *d*_r_ were negligibly small, indicating the absence of severe damage by the blunt spherical indenter. Although *μ* could be constant under small loads, it tended to increase slowly as *F*_v_ increased, and a prominent increasing trend of *μ* was seen under large loads. This trend could be attributed to subsurface cracking, under which the fracture toughness was calculated based on the scratch methodology.

### 3.3. Characterization of Fracture Toughness by Microscratch Method

Fracture toughness *K*_c_ of a brittle solid was calculated by the scratch-based methodology based on linear elastic fracture mechanics (LEFM) for axisymmetric indenters [52,53]:

(7) $$\sigma_{h}=\frac{F_{h}}{A_{h}}=\frac{K_{c}}{\sqrt{\Lambda }},\Lambda =\frac{A_{h}}{2l_{p}}$$

where *σ*_h_ is the nominal strength, Λ is the nominal size, *A*_h_ and *l*_p_ are the horizontally projected load-bearing contact area and the perimeter of the fracture surface, respectively, as shown in Figure 5a for a spherical intender. And *l*_p_ was calculated for a spherical indenter:

(8) $$l_{p}=2R\arccos\frac{R-d_{p}}{R}$$

**Figure 15:**
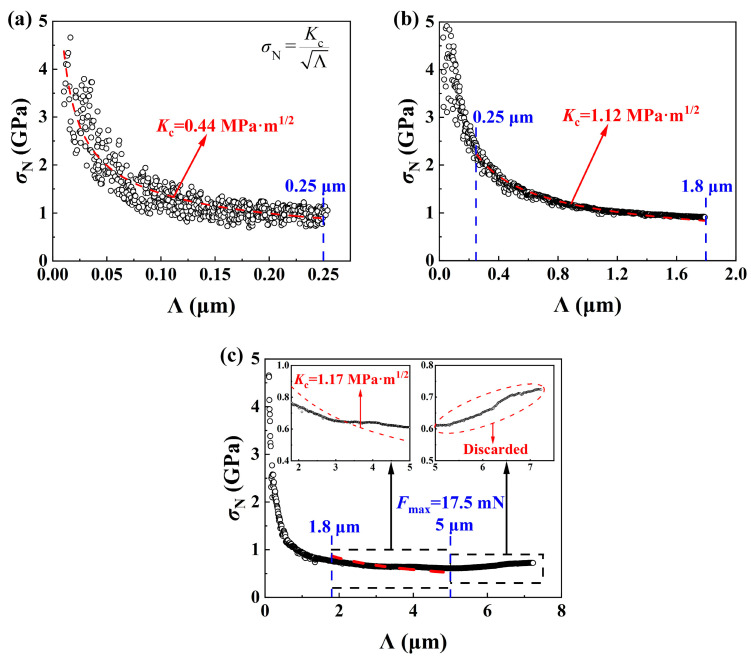
Analysis of fracture toughness *K*_c_ of SCAM using Equation (3) for scratching with a spherical intender under three different progressive loads: (**a**) 5~500 mN, (**b**) 5 mN~5 N, and (**c**) 5 mN~25 N.

Figure 15a–c show the variation of *σ*_N_ with Λ obtained by a spherical indenter with a radius of 500 μm under different progressive loads of 5~500 mN, 5 mN~5 N, and 5 mN~25 N. *K*_c_ was obtained by curve fitting *σ*_N_ vs. Λ using Equation (3), when *σ*_N_ decreases with increasing Λ. Figure 15a shows that *K*_c_ obtained under Λ < 0.25 μm was 0.44 MPa·m^1/2^, which was smaller than the reasonable values since cracking was not developed under small loads by a blunt indenter, under which the predominate role of elastic-plastic deformation resulted in an almost constant level of scratch friction coefficient as shown in Figure 14a. Figure 15b shows that *K*_c_ obtained under 0.25 μm < Λ < 1.8 μm was about 1.1 MPa·m^1/2^, which was a reasonable value since the semi-circular horizontal crack plane in front of the indenter assumed in LEFM was well developed, which was demonstrated by the increase in scratch friction coefficient as shown in Figure 14b. Figure 15c shows that a *σ*_N_ vs. Λ curve could not be fitted well under a large Λ, and *σ*_N_ even tended to increase, which could be explained by noting the formation of complex cracking and surface damage under large loads, which made the assumption of a cracking plane in LEFM invalid. An appropriate fitting curve under a suitable range of a normal load was used for the characterization of fracture toughness by a scratch-based method in order to avoid either a crack-free or a severe damage situation.

### 3.4. Characterization of Fracture Toughness by Indenter-Induced Cracking

Fracture toughness of materials was also obtained by the indenter-induced cracking technique based on Griffith-Irwin equilibrium fracture mechanics, whose applicability is still under debate, since different materials exhibit different cracking systems (e.g., radial, median, and transverse cracks) under different loads. The semi-empirical expressions proposed for a Vickers indenter are listed in Table 1, and were multiplied by the correction factor *k*_B_ = *f*(4)/*f*(3) = 1.073 when applied to a Berkovich indenter [54], since values of fracture toughness measured by Vickers and Berkovich indenters are linearly dependent:

(9) $$f(n)=\sqrt{\frac{n/2}{1+\frac{n}{2\pi }\sin\frac{2\pi }{n}}}$$

where *n* is the number of radial cracks (*n* = 4 for a Vickers indenter; and *n* = 3 for a Berkovich indenter), and *H*_V_ was replaced by *H*_B_, which was calculated as the ratio of the applied load *F* over the actual imprint area *A*_B_:

(10) $$H_{B}=\frac{F}{A_{B}}=\frac{4\sqrt{3}}{9}\frac{F\sin\alpha }{a^{2}}\approx 0.7\frac{F}{a^{2}},\;\alpha =65.27{}^{\circ}$$

**Figure 16:**
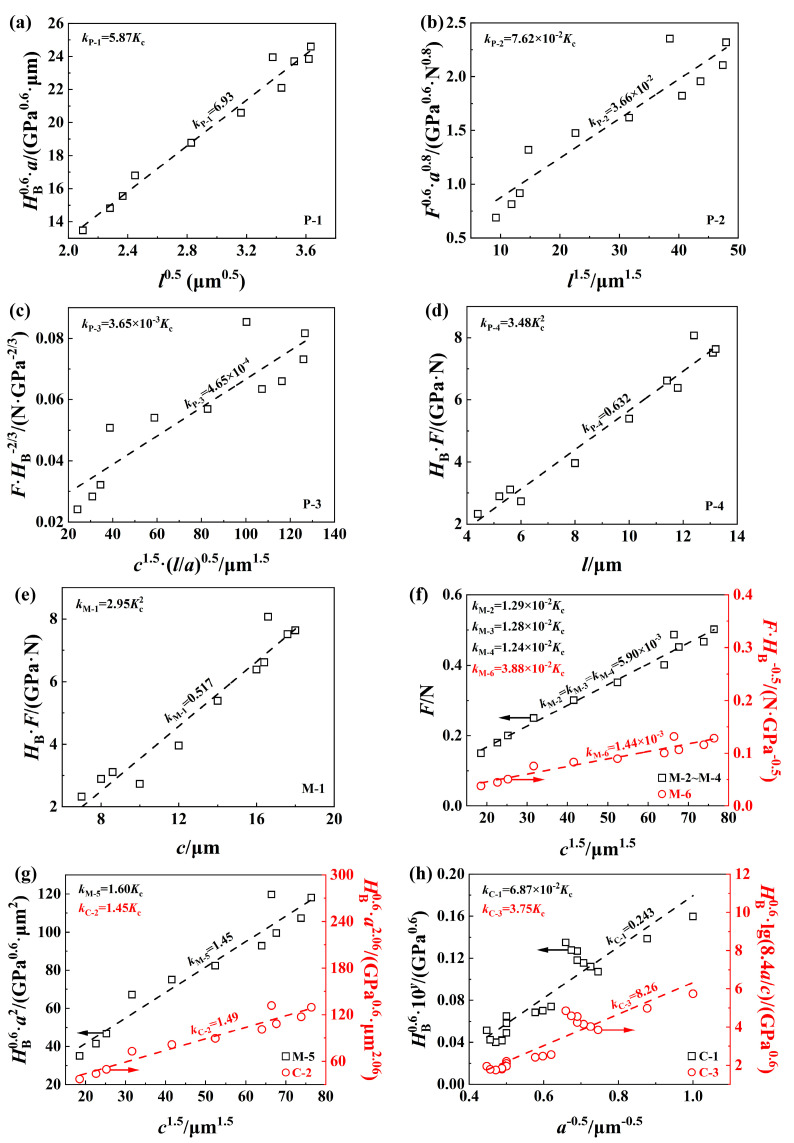
Determining fracture toughness (unit: MPa·m^1/2^) of SCAM by a Berkovich indenter based on the expressions listed in Table 1: (**a**) P-1; (**b**) P-2; (**c**) P-3; (**d**) P-4 (**e**) M-1; (**f**) M-2~M-4 and M-6; (**g**) M-5 and C-2; (**h**) C-1 and C-3.

Fracture toughness was obtained from the slope of the proportional fitting of the characteristic variables listed in Table 2, as shown in Figure 16. Radial cracks were invisible on the material surface under small loads (i.e., *F*_max_ ≤ 120 mN), and only data under large loads (i.e., *F*_max_ > 120 mN) were used for analysis. The *K*_c_ values calculated by the thirteen expressions listed in Table 1 are listed in Table 2, and only P-1, M-5, and C-2 gave reasonable values of *K*_c_ close to those obtained by the LEFM-based scratch method (i.e., 1.12 MPa·m^1/2^).

In general, low-toughness materials exhibit Palmqvist cracking, while medium cracking is found in materials with high toughness values. Actually, both types of cracking can be encountered in most materials [68], and a formal criterion based on the ratio *c*/*a* has been proposed to determine the type of cracking (i.e., Palmqvist cracking occurs when *c*/*a* < 2.5 and median cracking appears when *c*/*a* ≥ 2.5). The definitions of *c*, *a,* and *l* with the units of µm are shown in Figure 7d for the Berkovich indenter. It is understandable that almost all of the equations proposed for Palmqvist cracking underestimate the *K*_c_ value of SCAM under median cracking since *c*/*a* > 2.5 when *F*_max_ > 120 mN. Interestingly, C-2, which provides a reasonable *K*_c_ value of SCAM, has also provided a reasonable *K*_c_ value of an NaCl single crystal with a (100) cleavage plane [69] in previous research [66]. It is noteworthy that C-2 and M-5 have almost the same form, while M-5 has a more physical background than C-2, and the use of C-2 is not recommended due to the ambiguous exponent (i.e., −1.56) of (*c*/*a*). In brief, although P-1, M-5, and C-2 can give reasonable values of *K*_c_, only M-5 is recommended for SCAM, and it can be used as an optimal expression for calculating *K*_c_ values of other brittle and easily cleaved crystals.

## 4. Conclusions

Due to the easily cleaved nature of SCAM single crystals along its *c*-axis, it was challenging to obtain a high-quality surface via grinding and polishing, and the micromechanical properties of SCAM played a significant role in its ultra-precision polishing. Instrumented indentation with a Berkovich indenter and microscratch tests by spherical, Berkovich, and Vickers indenters were carried out on SCAM to study the deformation and damage under contact loading. As the load in nanoindentation increased, the damage became more and more severe, and micro-cleavages, cleavages, and radial cracks were observed. Elastic modulus and indentation hardness of SCAM obtained by nanoindentation were insensitive to indentation-induced damage under large, applied loads and were regarded as constant (i.e., *E*_IT_ = 226 GPa and *H*_IT_ = 12.1 GPa). Three different regimes were identified during scratching by Berkovich and Vickers indenters based on the morphologies of residual grooves, which were closely related to the variations and sudden changes of the scratch variables, including penetration depth, residual depth, friction coefficient, and acoustic emission. As the normal load increased during the scratch test, the damage became more and more severe, and debris, cracks, cleavages, leaf-shaped chips, and the associated step-like planes were found on the material’s surface. The blunt spherical indenter did not cause severe damage to the material under the loads applied in this study. Fracture toughness *K*_c_ = 1.12 MPa·m^1/2^ was obtained via scratch methodology with a spherical indenter based on linear elastic fracture mechanics. Fracture toughness was also obtained via the Vickers indenter-induced cracking technique using a Berkovich indenter, and an optimal expression was recommended for SCAM and other brittle and easily cleaved materials.

## Figures and Tables

**Table 1:** Expressions for calculating fracture toughness (unit: MPa·m^1/2^) of materials via indenter-induced cracking method using a Vickers indenter.

Equation	Expression	Crack Type
P-1	*K*_c_ = 0.0117(*l*/*a*)^−0.5^[*H*_V_/(*ΦE*)]^−0.4^*H*_V_*a*^0.5^ [55]	Palmqvist
P-2	*K*_c_ = 0.036 × 10^1.8^*E*^0.4^*F*^0.6^(2*a*)^−0.7^(*l*/*a*)^−1.5^ [56]	Palmqvist
P-3	*K*_c_ = 0.015(*a*/*l*)^0.5^(*E*/*H*_V_)^2/3^(*F*/*c*^1.5^) [57]	Palmqvist
P-4	*K*_c_ = *β* × 10^1.5^(*H*_V_*F*/4*l*)^0.5^ [58]	Palmqvist
M-1	*K*_c_ = (1 − 2*ν*)[(2*H*_V_/π)(*F*/c)]^0.5^/(2√2π^2^) [59]	Median
M-2	*K*_c_ = 72.5(*F*/*c*^1.5^) [60]	Median
M-3	*K*_c_ = 72.6(*F*/*c*^1.5^) [61]	Median
M-4	*K*_c_ = 75.2(*F*/*c*^1.5^) [62]	Median
M-5	*K*_c_ = 0.043(*c*/*a*)^−1.5^[*H*_V_/(*ΦE*)]^−0.4^*H*_V_*a*^0.5^ [63]	Median
M-6	*K*_c_ = 16[*E*/*H*_V_]^0.5^(*F*/*c*^1.5^) [64]	Median
C-1	*K*_c_ = 10*^y^*[*H*_V_/(*ΦE*)]^−0.4^*H*_V_*a*^0.5^ [65]	Both
C-2	*K*_c_ = 0.0473(*c*/*a*)^−1.56^[*H*_V_/(*ΦE*)]^−0.4^*H*_V_*a*^0.5^ [66]	Both
C-3	*K*_c_ = 0.0183lg(8.4*a*/*c*)[*H*_V_/(*ΦE*)]^−0.4^*H*_V_*a*^0.5^ [67]	Both

Notes: The expressions that are applicable to Palmqvist cracks, median cracks, and both Palmqvist and median cracks are indicated by P, M, and C, respectively. *H*_V_ and *E* are Vickers hardness and elastic modulus with units of GPa. *E* = 226 GPa for SCAM is obtained by nanoindentation, see Figure 6b. *F* is the normal load with the unit of N. *Φ* = 3 is a constant factor. *ν* (= 0.2 for SCAM) is Poisson’s ratio of the indented material. Based on Vickers indenter: *β* = 1/[3π(1–*ν*^2^)(√2tan*ψ*)], where *ψ* = 68°; and *y* = −1.59 − 0.34*x* − 2.02*x*^2^ + 11.23*x*^3^ − 24.97*x*^4^ + 15.32*x*^5^, where *x* = lg(*c*/*a*).

**Table 2:** Fracture toughness *K*_c_ (MPa·m^1/2^) of SCAM determined by a Berkovich indenter.

Equation	P-1	P-2	P-3	P-4	M-1	M-2	M-3	M-4	M-5	M-6	C-1	C-2	C-3
*K*_c_	**1.18**	0.48	0.13	0.43	0.42	0.46	0.46	0.48	**0.91**	0.37	3.54	**1.03**	3.75

Note: Bold figures indicate that fracture toughness values calculated are close to the one obtained by a spherical indenter (i.e., 1.12 MPa·m^1/2^) based on LEFM.

## Data Availability

The data presented in this study are available on request from the corresponding author. (due to privacy).
